# The Effects of Early Bispectral Index to Predict Poor Neurological Function in Cardiac Arrest Patients: A Systematic Review and Meta-Analysis

**DOI:** 10.3390/diagnostics10050271

**Published:** 2020-04-30

**Authors:** Chun-Yu Chang, Chien-Sheng Chen, Yung-Jiun Chien, Po-Chen Lin, Meng-Yu Wu

**Affiliations:** 1 School of Medicine, Tzu Chi University, Hualien 970, Taiwan; paulchang1231@gmail.com; 2 Department of Emergency Medicine, Taipei Tzu Chi Hospital, Buddhist Tzu Chi Medical Foundation, New Taipei 231, Taiwan; holeyeye@yahoo.com.tw (C.-S.C.); taipeitzuchier@gmail.com (P.-C.L.); 3 Department of Emergency Medicine, School of Medicine, Tzu Chi University, Hualien 970, Taiwan; 4 Department of Physical Medicine and Rehabilitation, Taipei Tzu Chi Hospital, Buddhist Tzu Chi Medical Foundation, New Taipei 231, Taiwan; jessica.kan.48@gmail.com

**Keywords:** bispectral index system, cardiac arrest, neurologic outcome, cerebral performance category, meta-analysis

## Abstract

The diagnostic performance of the bispectral index (BIS) to early predict neurological outcomes in patients achieving return of spontaneous circulation (ROSC) after cardiac arrest (CA) remained unclear. We searched PubMed, EMBASE, Scopus and CENTRAL for relevant studies through October 2019. Methodologic quality was assessed using the Quality Assessment of Diagnostic Accuracy Studies-2 (QUADAS-2) tool. Meta-analysis was performed using a linear mixed-effects model to the log-transformed data with a logistic distribution assumption. Bivariate meta-regression was performed to explore heterogeneity. In total, 13 studies with 999 CA adult patients were included. At the optimal threshold of 32, BIS obtained within 72 h of ROSC elicits a pooled sensitivity of 84.9% (95% confidence interval (CI), 71.1% to 92.7%), a pooled specificity of 85.9% (95% CI, 71.2% to 93.8%) and an area under the curve of 0.92. Moreover, a BIS cutoff < 12 yielded a pooled specificity of 95.0% (95% CI, 77.8% to 99.0%). In bivariate meta-regression, the timing of neurological outcome assessment, the adoption of targeted temperature management, and the administration of sedative agents or neuromuscular blocking agents (NMBA) were not identified as the potential source of heterogeneity. BIS retains good diagnostic performance during targeted temperature management (TTM) and in the presence of administrated sedative agents and NMBA. In conclusion, BIS can predict poor neurological outcomes early in patients with ROSC after CA with good diagnostic performance and should be incorporated into the neuroprognostication strategy algorithm.

## 1. Introduction

Sudden cardiac arrest (CA) is a challenge in emergency departments. Despite emergency medical services (EMS) and team-based cardiopulmonary resuscitation interventions, the mortality rates remain high. The survival discharge rate in out-of-hospital cardiac arrest (OHCA) patients has not exceed 5% in most communities [1,2]. In patients achieving return of spontaneous circulation (ROSC) after CA, 11–12% of them remain in a persistent coma status and up to 18% have moderate to severe functional impairment at hospital discharge [3,4,5,6]. In post-cardiac arrest syndrome, the ischemic reperfusion injury and post-anoxic brain injury are two major causes of mortality in severe neurological damage [7,8]. Current guidelines recommend neuroprognostication in patients who remain comatose and unresponsive to pain stimulus after 72 h of ROSC [9]. Several clinical examinations or tools have been suggested to aid in neuroprognostication, such as bilateral absence of pupillary and corneal reflexes, bilateral absence of N20 short-latency somatosensory evoked potentials wave, and a set of specific features on electroencephalography (EEG), brain imaging, etc.

The bispectral index system (BIS) is a non-invasive technology to measure brain activity by analysis of EEG [10]. BIS is widely used for determining the depth of anesthesia to prevent intraoperative awareness during anesthesia. BIS values range from 0 to 100; BIS of 0 represents flat-line EEG reflecting no brain activity, while 100 represents an intact memory and wakefulness [11]. In several studies, BIS was used to predict neurological outcomes in cardiac arrest patients. However, there is uncertainty as to whether BIS is a reliable tool to predict neurological outcomes in patients achieving ROSC after CA. In addition, there is a lack of strong evidence to confirm the results. In this meta-analysis, we hypothesized that early predicted neurological outcomes based on BIS are reliable in patients achieving ROSC after CA.

## 2. Methods

### 2.1. Study Design

This is a meta-analysis of eligible studies aimed at investigating the application of BIS to predict poor neurological outcomes in the cardiac arrest population. This study complies with the recommendations made by the Preferred Reporting Items for a Systematic Review and Meta-analysis of Diagnostic Test Accuracy Studies (The PRISMA-DTA) statement [12].

### 2.2. Search Strategy

Two authors (Y.-J.C. and C.-Y.C.) searched PubMed, EMBASE, Cochrane Central Register of Controlled Trials (CENTRAL) and Scopus databases. Mesh terms from PubMed and Emtree terms from Embase were used in combination with free-text words. The Boolean operator “OR” was used to cover similar concepts while the Boolean operator “AND” was used to intersect different concepts. The following terms were used to search for BIS using the “OR” operator: “bispectral index” OR “bispectral index score” OR “bispectral index monitoring” OR “consciousness monitors.” Likewise, the following terms were used to search for cardiac arrest using the “OR” operator: “cardiac arrest” OR “heart arrest” OR “out-of-hospital cardiac arrest” OR “cardiopulmonary resuscitation” OR “resuscitation” OR “return of spontaneous circulation.” The search results of BIS and cardiac arrest were intersected using the “AND” operator. The relevant studies published through October 2019 were analyzed without language or geographical limitations, and were screened by titles, abstracts, and full texts from the electronic databases. The reference lists of the primary studies and relevant reviews were also used to search additional studies. 

### 2.3. Eligibility Criteria

All studies identified from electronic databases were screened and selected by two authors (Y.-J.C. and C.-Y.C.) independently, as per the following inclusion criteria: (a) studies of all design investigating the application of BIS obtained within 72 h of ROSC to predict neurological outcomes except for letters, case reports, editorials or reviews; (b) adult populations with cardiac arrest presenting to the emergency department or inpatient settings; (c) limited to human studies and no language or ethnicity restrictions were applied. Studies were excluded if they did not meet the inclusion criteria.

### 2.4. Index and Reference Tests

We defined the index test as BIS score obtained in patients with ROSC after cardiac arrest. The optimal cutoff value for a positive test result has not been defined and thus depends on the primary studies where the optimal cutoff was chosen based on a specified criterion. We defined the reference test as Cerebral Performance Category (CPC) scale [13], which ranges from 1 to 5. CPC 1 indicates good cerebral performance, CPC 2 indicates moderate cerebral disability, CPC 3 indicates severe cerebral disability, CPC 4 indicates persistent vegetative state, and CPC 5 indicates brain death or clinical death. For statistical analysis and in line with the Utstein report for OHCA [14], CPC 1 and 2 are considered good neurological outcomes, whereas CPC 3, 4, and 5 are considered poor neurological outcomes.

### 2.5. Risk of Bias in Individual Studies

Two authors (Y.-J.C. and C.-Y.C.) evaluated the methodological quality of all included studies independently by using the Quality Assessment of Diagnostic Accuracy Studies-2 (QUADAS-2). The third author (M.-Y.W.) provided the consensus or discussion for disagreements.

### 2.6. Data Extraction

The information of included studies was extracted by two authors independently (Y.-J.C. and C.-Y.C.) and included the authors, publication year, country, settings, study design, number of patients, characteristics of the patients and BIS monitoring, prognosis assessment, optimal cutoff BIS value to predict neurological outcomes, criteria based on which the optimal cutoff was chosen, and quantitative data required to construct a standard diagnostic test 2 × 2 table. In addition, based on current concepts from the International Liaison Committee on Resuscitation (ILCOR) advisory committee, the American Heart Association (AHA), and the European Resuscitation Council (ERC), TTM reduced post-anoxic injury and improving neurological outcomes after cardiac arrest is the only neuroprotective intervention currently recommended in the cardiac arrest population. During TTM, targeted temperatures that have been recommended range from 32 °C to 36 °C. Therefore, regimen of TTM was also extracted and analyzed. All available pairs of sensitivity and specificity at the corresponding threshold were extracted. In studies where BIS was used to predict good neurological outcomes, we reconstructed these data into predicting poor neurological outcomes.

### 2.7. Statistical Analysis

From the included primary studies, we calculated the pooled sensitivity and specificity at the optimal threshold by the method proposed by Steinhauser et al. [15] Conventionally, the bivariate model [16] has been widely adopted to calculate the pooled sensitivity and specificity. The bivariate model takes into consideration the expected trade-off in sensitivity and specificity. However, it has some limitations. First, in a study where multiple pairs of sensitivity and specificity at different thresholds were reported, only one pair of them could be selected and entered into the model. Most of the time, such a selection is based on the Youden index, which is a commonly used summary measure of the ROC curve for accuracy and evaluation of the overall discriminative power of a diagnostic test that yields the greatest combination of sensitivity and specificity (calculated as sensitivity + specificity – 1, range from 0 for a poor accuracy to 1.0 for a perfect test), leading to a too optimistic an evaluation of the index tests [17]. Second, in the circumstances where the thresholds differed across the primary studies, although the bivariate model could estimate the pooled sensitivity and specificity, this is clinically unhelpful due to the notional unspecified average of the thresholds and should thus be avoided [18]. With the method proposed by Steinhauser et al., multiple pairs of sensitivity and specificity at the corresponding thresholds in each primary study could be taken into consideration. The pooled sensitivity and specificity along with a summary receiver operating characteristic (SROC) curve could be derived and the optimal threshold could be determined [15]. 

In brief, in analogy to the logit transformation in the bivariate model, we log-transformed the threshold data with a logistic distribution assumption. We then synthesized our data using all the linear mixed-effects models with a different random effects structure and set the weighting parameter as 0.5. To choose the model, we selected the one with the smallest restricted maximum likelihood (REML) criterion. The optimal threshold was determined by the threshold that maximizes the Youden index. When the 2 × 2 tables contained zero cells, we applied continuity correction by adding 0.5 to each cell. In order to calculate between-study heterogeneity and explore the reasons accounting for it, we selected the threshold that maximizes the Youden index in studies where multiple thresholds were reported, limiting only one threshold for each study. Between-study heterogeneity was quantified using the Chi-squared test. Correlation of sensitivities and false positive rates were calculated. Bivariate meta-regression modeled with different covariates, one at each time, was conducted to explore whether the timing of outcome assessment, the adoption of TTM after ROSC, and the administration of sedative agents and neuromuscular blocking agents (NMBA) account for the heterogeneity. We a priori dichotomized the timing of the outcome assessment to within 30 days and beyond 30 days based on the evidence that the condition of the neurological outcome relatively stabilized after one month [19]. Two different null hypotheses were tested. First, we tested the hypothesis that these covariates do not explain variance in the logit-transformed sensitivities and specificities, by testing the regression coefficient for the sensitivity and specificity, respectively. Second, we tested the hypothesis that these covariates do not explain variance in the logit-transformed pairs of sensitivity and specificity by performing likelihood-ratio tests to compare the fit of the two bivariate meta-regression models, i.e., a model with intercept only and a model with covariate, one at a time. The publication bias was examined using Deek’s funnel plot [20]. A *p*-value < 0.1 of the regression test indicates significant publication bias. Statistical analysis was conducted using R version 3.6.0 [21] (Foundation for Statistical Computing) with “diagmeta” [22], “mada” [23], and “metafor” [24] packages. A *p* value < 0.05 was considered statistically significant.

## 3. Results

### 3.1. Study Identification and Selection

**Figure 1:**
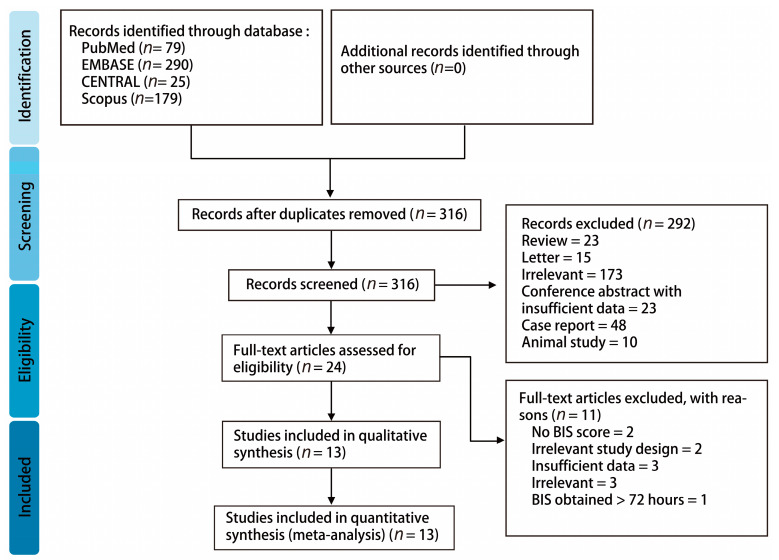
The detailed PRISMA flow diagram.

A total of 573 studies were identified from major databases, including PubMed (*n* = 79), EMBASE (*n* = 290), Scopus (*n* = 179), and CENTRAL (*n* = 25). After removing 257 duplicates, the remaining studies were screened for eligibility. A total of 292 of them were excluded, owing to their lack of relevance, animal studies, insufficient data, or other article types. As a result, 24 studies were assessed with full-text review. A total of 11 articles were excluded due to an irrelevant outcome, insufficient data, lack of a BIS score, an irrelevant study design, and BIS obtained within 72 h of ROSC. Finally, 13 studies involving 999 patients were used for final analysis. The detailed Preferred Reporting Items for a Systematic Review (PRISMA) flow diagram is shown in Figure 1.

### 3.2. Study Characteristics

**Figure 2:**
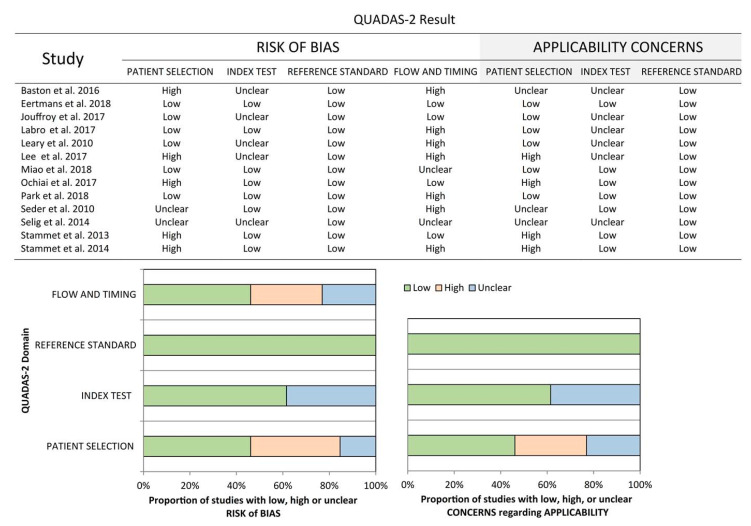
The quality and risk of bias assessment of the included studies. QUADAS-2: Quality Assessment of Diagnostic Accuracy Studies-2.

Thirteen studies with a total of 999 patients were included. Among them, 10 are prospective studies [25,26,27,28,29,30,31,32,33,34], 2 are retrospective [35,36], and the study design was not mentioned in 1 study [37]. Three of them were conference abstracts with sufficient information for analysis [31,32,35]. Seven studies were conducted in Europe [25,28,31,32,33,34,37], two in North America [26,30], and four in Asia [27,29,35,36]. Three studies included only OHCA patients [25,27,28], while the rest included all CA patients. Most of the studies adopted TTM after ROSC except for two studies [29,31]. Only some of the patients had TTM in the Selig et al. study [37]. Sedative agents were used in eight studies [25,26,28,30,32,33,34,36], and NMBA was used in 10 studies [25,26,27,28,30,32,33,34,35,36]. In studies where the data were originally reported as predicting good neurological outcomes (CPC 1–2) [27,30], we manually reconstructed the data into a 2 × 2 table predicting the poor neurological outcomes (CPC 3–5). In studies where the data were originally reported as predicting survival [29,30], we regarded them as predicting neurological outcomes of CPC 1–4, and then manually reconstructed the data into a 2 × 2 table predicting the neurological outcome of CPC 5. The timing when the prognosis assessment was performed varied across studies. For the convenience of statistical analysis, the Leary et al. study was separated by the outcomes to be predicted into the Leary et al.-A study and the Leary et al.-B study. The study characteristics were presented in detail in Table 1. The QUADAS-2 scores were used to evaluate the quality of the included studies. The analysis of the risk of bias in each of the included studies is listed in Figure 2. Most information is derived from the studies at moderate risk of bias.

### 3.3. Overall Meta-Analysis of BIS for the Prediction of Poor Neurological Outcomes

#### 3.3.1. Descriptive Data for the Included Studies

**Figure 3:**
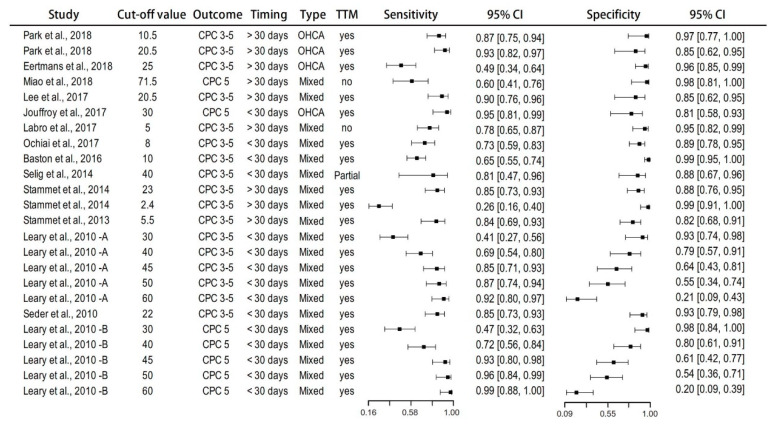
Sensitivity and specificity of the included studies.

**Figure 4:**
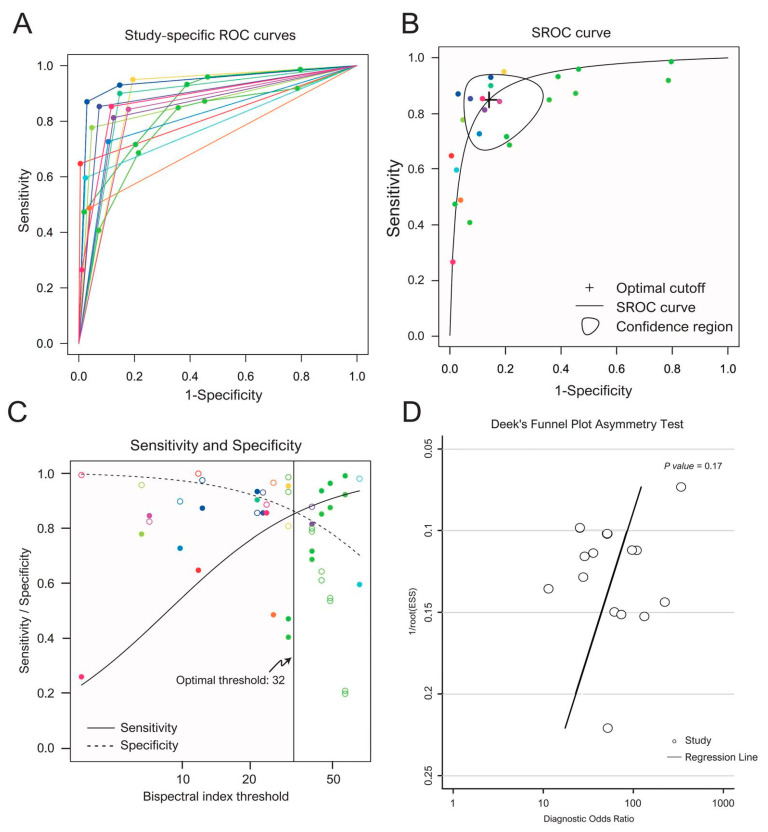
(**A**) Study-specific receiver operating characteristic (ROC) curves. (**B**) Summary receiver operating characteristic (SROC) curve. The points represent the pair of sensitivity and specificity at a given threshold in each included study. Points of same color belong to the same primary study. The cross mark represents the pooled sensitivity and specificity at the optimal cutoff (BIS = 32). (**C**) Trade-off of sensitivity and specificity. The pairs of open circle and filled circle represent the sensitivity and specificity at a given threshold. Circles of same color belong to the same study at different given thresholds. The vertical solid line represents the optimal threshold. (**D**) Deek’s funnel plot. The regression test showed no significant publication bias (*p* = 0.17).

The forest plot of the sensitivity and specificity of each included study is presented in Figure 3. Study-specific ROC curves are presented in Figure 4A. 

#### 3.3.2. Pooled Sensitivity, Specificity and Optimal Threshold

We chose the “different random intercepts and common random slope” model because it minimized the REML criterion. As shown in Figure 4B, the optimal threshold for BIS values to predict poor neurological outcomes was 32, with the pooled sensitivity of 84.9% (95% CI, 71.1% to 92.7%) and the pooled specificity of 85.9% (95% CI, 71.2% to 93.8%). The area under the curve (AUC) was 0.92. Moreover, a BIS cutoff < 12 yielded a pooled specificity of 95.0% (95% CI, 77.8% to 99.0%). The trade-off of the sensitivity and specificity as the threshold varies is presented in Figure 4C. As the threshold of BIS value decreased, the specificity increased while the sensitivity decreased, and vice versa.

### 3.4. Exploration of the Potential Sources of Heterogeneity

The between-study heterogeneity for both sensitivities (Chi-squared = 56.3; *p* < 0.001) and specificities (Chi-squared = 55.6; *p* < 0.001) were significant. The Pearson correlation coefficient of sensitivities and false positive rates was 0.59. This may explain some of the heterogeneity between primary studies. In addition, whether the difference in the CA type, the outcome to be predicted, the timing of the outcome assessment, the use of TTM, and the administration of sedative agents and NMBA also explain the heterogeneity is unknown. Hence, we conducted bivariate meta-regression modeled with different covariates one at a time. The likelihood-ratio test revealed no significant difference in the fit of the models with these covariates and without (Table 2).

### 3.5. Publication Bias

Deek’s funnel plot asymmetry test was conducted to explore the potential publication bias, as shown in Figure 4D. The regression test showed no significant publication bias (*p* = 0.17).

## 4. Discussion

BIS is a simplified EEG system and is conventionally designed for monitoring the depth of anesthesia during surgery. Over the past decade, BIS has also been evaluated as an early prognostic tool for neurological outcomes in patients achieving ROSC after CA. However, the optimal cutoff value of BIS to predict poor neurological outcomes has not been determined and has ranged from 5 to 71.5 in previous studies [29,31]. As a result, it remains challenging for physicians to apply BIS to predict neurological outcomes in clinical practice. The present meta-analysis investigated the overall diagnostic performance of BIS to predict poor neurological outcomes early in patients with ROSC after CA and observed that it is a useful tool. The AUC is 0.92 and the optimal cutoff value of BIS < 32 predicted poor neurological outcomes with the pooled sensitivity of 84.9% (95% CI, 71.1% to 92.7%) and the pooled specificity of 85.9% (95% CI, 71.2% to 93.8%). Moreover, the cutoff value of BIS < 12 yielded the pooled specificity of 95.0% (95% CI, 77.8% to 99.0%) with the false positive rate of 5.0%.

The optimal timing for applying BIS to predict poor neurological outcomes in patients with ROSC after CA is still unclear. In the statement of the 2015 joint guidelines of the ERC and the European Society of Intensive Care Medicine (ESICM), the neuroprognostication is applicable no earlier than 72 h after ROSC in comatose patients with a Glasgow Coma Scale (GCS) Motor score less than 2, should all the major confounders have been excluded [9,38]. The reason for the timing of neuroprognostication is based on the fact that the duration of brain recovery is completed within 72 h after global post-anoxic injury [39,40]. However, the recommendation is based on weak evidence. In the included studies of the present meta-analysis, BIS assessment was performed within 72 h of ROSC and had the AUC of 0.92 and the false positive rate of 5% at the cutoff of BIS < 12.

TTM provided neuroprotective effects by slowing the cellular metabolism to prevent progressive cell apoptosis and reduce reperfusion injury, and is the class I recommendation in comatose adult patients after ROSC [41]. However, the reliability of several neurological assessment tools was impaired during TTM itself and also by the sedatives and the NMBA used to maintain it. In the meta-regression analysis, we found no significant differences in the diagnostic performance of BIS in patients treated with TTM, sedatives, and NMBA as compared with those who were not. This suggests that BIS remains reliable during TTM and during the use of sedatives and NMBA. On the other hand, previous studies reported that 27% of post-hypoxic coma patients regained consciousness in 28 days and 9% remained in a comatose state [19,42]. In the Rüdiger Pfeifer et al. [43] prospective clinical study, 18.6% of ROSC patients remained in a persistent vegetative state after 28 days. These results indicated that the condition of neurological damage due to ischemia reperfusion injury stabilized after one month. However, in the meta-regression analysis, we did not observe significant differences in the diagnostic performance of BIS between patients whose CPC assessment was performed within one month and those whose CPC assessment was performed beyond one month. 

The between-study heterogeneity for both sensitivity and specificity were significant and may arise from the following sources. First, the heterogeneity is most likely attributed to the negative correlation of the sensitivity and specificity as the optimal threshold of BIS differed across the included studies. Second, the BIS value selected for predicting neurological outcomes differed across studies. For instance, four studies used the mean BIS value [25,28,36,37], while others used the maximal BIS value [29], the lowest BIS value [34], the sustained plateau values [26], and the BIS value at a specific time point [30,33]. Third, although all the included studies obtained the BIS values within 72 h of ROSC, the timing of the initiation of BIS still differed across studies. Fourth, TTM, sedative agents and NMBA were used in some studies but not in others. Although the meta-regression analysis suggested that these factors were not likely to account for the heterogeneity, the relatively low study number and unbalanced study number may bias the results.

Our study has several limitations. First, most of the included studies are prospective observational studies but three articles are abstracts with limited information in the BIS monitoring system, the selection of the BIS values for analysis, and the detailed patient characteristics [31,32,35]. Second, the selection of BIS values for predicting neurological outcomes and the timing of applying BIS monitors differed across studies. Third, all types of cardiac arrest have been analyzed, but only three studies provided detailed information. This may be an important potential confounder for BIS prediction of neurological outcomes in different types of cardiac arrest population. In addition, many factors may impair the prediction function of BIS in OHCA patients, including sedation and analgesia. In current clinical practice, administration of sedation and analgesia in OHCA patients was individual and there was no detailed protocol for physicians. The detailed information was only reported in a few included studies. Finally, there are few reported studies focused on BIS in the cardiac arrest population. In our meta-analysis, only 13 reported articles met our inclusion criteria. In future, this result is required to be confirmed by a large randomized control trial.

## 5. Conclusions

In summary, BIS obtained within 72 h of ROSC in patients after CA can predict poor neurological outcomes with good diagnostic performance, with the pooled sensitivity of 84.9%, the pooled specificity of 85.9%, and the AUC of 0.92 at the optimal threshold of 32. Moreover, a BIS cutoff < 12 yielded a pooled specificity of 95.0%. We suggest that BIS may be incorporated into the neuroprognostication strategy algorithm along with other currently recommended tools.

## Figures and Tables

**Table 1:** Study characteristics.

Study	Country	Design	Population	Therapeutic Hypothermia	BIS Initiation	Age ^†^	Male (%)	Outcome	Timing	Threshold	N	TP	FP	FN	TN
Jouffroy et al., 2017 [25]	France	Prospective cohort	Refractory OHCA	32–34 °C during the first 12–24 h	During TTM in the first 12–24 h	52.0(13.0)	30(65.2)	CPC 5	Day 28	30	46	28	3	1	14
Selig et al., 2014 [37]	Germany	Not mentioned	OHCA, IHCA	Mild hypothermia was induced for 12–24 h in 47.5% of the patients with ROSC	On average 37.0 min after the initiation of CPR	69 (21–91) ^‡^	58(73.4)	CPC 3–5	Day 3, 7 and 1 month	40	26	6	2	1	17
Seder et al., 2010 [26]	USA	Prospective cohort	Patients with HIE within 12 h of ROSC after CA	Targeted bladder temperature of 33 °C for 18–24 h	After the first dose of NMBA (median 84 (45–166) minutes after TH)	62(48–72) ^§^	54(65.0)	CPC 3–5	At hospital discharge	22	83	43	2	7	31
Park et al., 2018 [27]	South Korea	Prospective cohort	OHCA	Core temperature of 32–34 °C for 24 h, followed by an increase of 0.25 °C/hour to 36.5 °C	Average time from ROSC to the first significant BIS value = 2.3 ± 1.0 h	55.6(16.8)	49(75.4)	CPC 3–5	6 months	10.5	65	43	0	6	16
								CPC 3–5 ^Φ^	6 months	20.5	65	46 ^Φ^	2 ^Φ^	3 ^Φ^	14 ^Φ^
Eertmans et al., 2018 [28]	Belgium	Prospective cohort	OHCA	TTM at 33 °C for 24 h followed by rewarming for 12 h (0.3 °C/hour)	Continuous monitoring for 36 h during hypothermia and rewarming phase	Good outcome: 67.0(13.0) Poor outcome: 61.0(13.0)	Good outcome: 31(81.6) Poor outcome: 31(79.5)	CPC 3–5	Day 180	25	77	19	1	20	37
Miao et al., 2018 [29]	China	Prospective cohort	Patients resuscitated from CA	Not mentioned	On admission to the ICU, continuous monitoring for 12 h	65.0 (20.0)	27(60.0)	CPC 5*	Day 60	71.5	45	15 *	0 *	10 *	20 *
Lee et al., 2017 [35]	South Korea	Retrospective cohort	CA	TTM	As soon as TTM started in CA patients	Not mentioned	Not mentioned	CPC 3–5	6 months	20.5	50	31	2	3	14
Leary et al., 2010 – A [30]	USA	Prospective cohort	Patients who achieved ROSC after resuscitation from OHCA and IHCA	Temperature of 32–34 °C for 24 h	Immediately after resuscitation	55.0(16.0)	36(58.1)	CPC 3–5 ^Φ^	At hospital discharge	30	62	17 ^Φ^	1 ^Φ^	25 ^Φ^	19 ^Φ^
								CPC 3–5 ^Φ^	At hospital discharge	40	62	29 ^Φ^	4 ^Φ^	13 ^Φ^	16 ^Φ^
								CPC 3–5 ^Φ^	At hospital discharge	45	62	36 ^Φ^	7 ^Φ^	6 ^Φ^	13 ^Φ^
								CPC 3–5 ^Φ^	At hospital discharge	50	62	37 ^Φ^	9 ^Φ^	5 ^Φ^	11 ^Φ^
								CPC 3–5 ^Φ^	At hospital discharge	60	62	39 ^Φ^	16 ^Φ^	3 ^Φ^	4 ^Φ^
Leary et al., 2010 – B [30]	USA	Prospective cohort	Patients who achieved ROSC after resuscitation from OHCA and IHCA	Temperature of 32–34 °C for 24 h	Immediately after resuscitation	55.0(16.0)	36(58.1)	CPC 5 *	At hospital discharge	30	62	17 *	0 *	19 *	26 *
								CPC 5 *	At hospital discharge	40	62	26 *	5 *	10 *	21 *
								CPC 5 *	At hospital discharge	45	62	34 *	10 *	2 *	16 *
								CPC 5 *	At hospital discharge	50	62	35 *	12 *	1 *	14 *
								CPC 5 *	At hospital discharge	60	62	36 *	21 *	0 *	5 *
Labro et al., 2017 [31]	France	Prospective cohort	Patients admitted to the ICU for CA	Not mentioned	Mean duration from ROSC to BIS measurement = 5.7 ± 3.0 h	57.6(16.8)	61(70.9)	CPC 3–5	3 months	5	86	43	1	12	30
Ochiai et al., 2017 [36]	Japan	Retrospective cohort	Consecutive adult patients with OHCA or IHCA	The target temperature (33 or 34 °C), the period of maintaining this temperature (24 or 48 h), and rewarming times (12–48 h) varied depending on the era of patient treatment.	Recorded at intervals not exceeding 2 h between the time of target temperature achievement and completion of rewarming	Patients without clinical seizure: 59(47–68) ^§^ Patients with clinical seizure: 55(43–68) ^§^	Patients without clinical seizure: 51(70.8) Patients with clinical seizure: 23(74.2)	CPC 3–5	Day 30	68	103	38	5	14	46
Baston et al., 2016 [32]	Spain	Prospective cohort	Successfully resuscitated patients who were unconscious at arrival	TH for 24 h	Started after TH, continuously monitor for 48 h	73.8	140(75.2)	CPC 3–5	At hospital discharge	10	185	61	0	33	91
Stammet et al., 2014 [33]	Luxembourg	Prospective cohort	All successfully resuscitated adult CA patients	Induced hypothermia at 33 °C for 24 h	Started after hypothermia and throughout the 24 h period	Good outcome: 57(21–81) ^‡^ Poor outcome: 67(24–83) ^‡^	Good outcome: 42(91%) Poor outcome: 36(72%)	CPC 3–5	6 months	23	96	43	5	7	41
								CPC 3–5	6 months	2.4	96	13	0	37	46
Stammet et al., 2013 [34]	Luxembourg	Prospective cohort	CA patients admitted to the general ICU of the hospital	Patients were treated with hypothermia at 33 °C for 24 h after successful resuscitation	After admission to the ICU, monitor for 48 h	Good outcome: 61(29–82) ^‡^ Poor outcome: 69(38–83) ^‡^	Good outcome: 34(82.9) Poor outcome: 23(67.6)	CPC 3–5	6 months	5.5	75	29	7	5	34

^†^ Presented as mean (SD) unless specified otherwise. ^‡^ Presented as median (range). ^§^ Presented as median (interquartile range). ^Φ^ Data were originally reported as predicting good neurological outcomes (CPC 1–2) and were reconstructed to predict poor neurological outcomes (CPC 3–5). * Data were originally reported as predicting survival (which could be regarded as CPC 1–4) and were reconstructed to predict neurological outcomes of CPC. BIS: bispectral index system; N: sample size; TP: true positive; FP: false positive; FN: false negative; TN: true negative; CPC: cerebral performance category; OHCA: out-of-hospital cardiac arrest; IHCA: in-hospital cardiac arrest; CPR: cardiopulmonary resuscitation; CA: cardiac arrest; HIE: hypoxic-ischemic encephalopathy; ROSC: return of spontaneous circulation; NMBA: neuromuscular blocking agents; TH: therapeutic hypothermia; TTM: targeted temperature management; EMG: electromyography; ICU: intensive care units; CCU: coronary care unit; EICU: emergency intensive care unit.

**Table 2:** Bivariate meta-regression models with different covariates.

Covariate	Sensitivity (95% CI)	*p* Value ^†^	Specificity (95% CI)	*p* Value ^‡^	*p* Value ^§^
**CA type**					0.84
OHCA	78.5% (58.1–90.6%)	<0.01	91.4% (72.7–97.7%)	<0.01	
Mixed	81.2% (72.5–87.6%)	<0.01	87.2% (78.5–92.8%)	<0.01	
**Outcome**					0.65
CPC 3–5	79.3% (70.5–86.0%)	<0.01	89.2% (81.5–93.9%)	<0.01	
CPC 5	84.7% (66.3–94.0%)	<0.01	82.6% (58.7–94.1%)	<0.01	
**Timing**					0.37
< 30 days	83.0% (72.4–90.1%)	<0.01	83.7% (71.2–91.5%)	<0.01	
> 30 days	77.9% (66.0–86.4%)	<0.01	91.6% (83.1–96.0%)	<0.01	
**TTM**					0.26
Yes	82.2% (74.0–88.2%)	<0.01	86.1% (77.3–91.9%)	<0.01	
No	69.8% (43.7–87.3%)	0.13	96.7% (82.4–99.5%)	<0.01	
**Sedation**					0.37
Yes	80.7% (70.9–87.8%)	<0.01	86.2% (76.3–92.4%)	<0.01	
No	81.1% (66.8–90.1%)	<0.01	92.8% (81.2–97.5%)	<0.01	
**NMBA**					0.43
Yes	82.1% (74.0–88.1%)	<0.01	86.2% (77.3–91.9%)	<0.01	
No	74.1% (52.5–88.1%)	0.03	94.0% (80.0–98.4%)	<0.01	

^†^ Test for sensitivity. ^‡^ Test for specificity. ^§^ Likelihood-ratio test comparing the fit of the model with versus without the covariate. CA: cardiac arrest; OHCA: out-of-hospital cardiac arrest; CPC: cerebral performance category; TTM: targeted temperature management; NMBA: neuromuscular blocking agents.

## Abbreviations

AHA	American Heart Association
AUC	Area under the curve
BIS	Bispectral index
CA	Cardiac arrest
CENTRAL	Cochrane Central Register of Controlled Trials
CPC	Cerebral Performance Category
CI	Confidence interval
EEG	Electroencephalography
EMS	Emergency medical services
ERC	European Resuscitation Council
ESICM	European Society of Intensive Care Medicine
GCS	Glasgow Coma Scale
ILCOR	International Liaison Committee on Resuscitation
NMBA	Neuromuscular blocking agents
OHCA	Out-of-hospital cardiac arrest
PRISMA-DTA	Preferred Reporting Items for a Systematic Review and Meta-analysis of Diagnostic Test Accuracy Studies
QUADAS-2	Quality Assessment of Diagnostic Accuracy Studies-2
REML	Restricted maximum likelihood
ROSC	Return of spontaneous circulation
SROC	Summary receiver operating characteristic
TTM	Targeted temperature management
